# Evaluation Criteria for Weight Management Apps: Validation Using a Modified Delphi Process

**DOI:** 10.2196/16899

**Published:** 2020-07-22

**Authors:** Noemí Robles, Elisa Puigdomènech Puig, Corpus Gómez-Calderón, Francesc Saigí-Rubió, Guillem Cuatrecasas Cambra, Alberto Zamora, Montse Moharra, Guillermo Paluzié, Mariona Balfegó, Carme Carrion

**Affiliations:** 1 eHealth Lab Research Group Universitat Oberta de Catalunya Barcelona Spain; 2 eHealth Center Universitat Oberta de Catalunya Barcelona Spain; 3 Red de Investigación en Servicios de Salud en Enfermedades Crónicas Barcelona Spain; 4 Agència de Qualitat i Avaluació Sanitàries de Catalunya Barcelona Spain; 5 Marina Salud Alicante Spain; 6 Faculty of Health Sciences Universitat Oberta de Catalunya Barcelona Spain; 7 Interdisciplinary Research Group on ICTs Universitat Oberta de Catalunya Barcelona Spain; 8 Clínica Sagrada Família CPEN SL Servei d'Endocrinologia i Nutrició Barcelona Spain; 9 Corporació de Salut del Maresme i la Selva Hospital de Blanes Blanes Spain; 10 Grup de Medicina Traslacional i Ciències de la Decisió Universitat de Girona Girona Spain; 11 Centro de Investigación Biomédica en Red en Epidemiología y Salud Pública Barcelona Spain

**Keywords:** mHealth, technology assessment, obesity, overweight, Delphi technique, consensus

## Abstract

**Background:**

The use of apps for weight management has increased over recent years; however, there is a lack of evidence regarding the efficacy and safety of these apps. The EVALAPPS project will develop and validate an assessment instrument to specifically assess the safety and efficacy of weight management apps.

**Objective:**

The aim of this study was to reach a consensus among stakeholders on a comprehensive set of criteria to guide development of the EVALAPPS assessment instrument. A modified Delphi process was used in order to verify the robustness of the criteria that had been identified through a literature review and to prioritize a set of the identified criteria.

**Methods:**

Stakeholders (n=31) were invited to participate in a 2-round Delphi process with 114 initial criteria that had been identified from the literature. In round 1, participants rated criteria according to relevance on a scale from 0 (“I suggest this criterion is excluded”) to 5 (“This criterion is extremely relevant”). A criterion was accepted if the median rating was 4 or higher and if the relative intraquartile range was equal to 0.67 or lower. In round 2, participants were asked about criteria that had been discarded in round 1. A prioritization strategy was used to identify crucial criteria according to (1) the importance attributed by participants (criteria with a mean rating of 4.00 or higher), (2) the level of consensus (criteria with a score of 4 or 5 by at least 80% of the participants).

**Results:**

The response rate was 83.9% (26/31) in round 1 and 90.3% (28/31) in round 2. A total of 107 out of 114 criteria (93.9%) were accepted by consensus—105 criteria in round 1 and 2 criteria in round 2. After prioritization, 53 criteria were deemed crucial. These related mainly to the dimensions of security and privacy (13/53, 24.5%) and usability (9/53, 17.0%), followed by activity data (5/53, 9.4%), clinical effectiveness (5/53, 9.4%), and reliability (5/53, 9.4%).

**Conclusions:**

Results confirmed the robustness of the criteria that were identified, with those relating to security and privacy being deemed most relevant by stakeholders. Additionally, a specific set of criteria based on health indicators (activity data, physical state data, and personal data) was also prioritized.

## Introduction

The prevalence of obesity and being overweight has nearly tripled over the last 30 years and appears likely to continue increasing in the near future [1]. Obesity and being overweight are considered risk factors for type 2 diabetes, cardiovascular diseases, musculoskeletal disorders, and some cancers [2]. As several factors are known to influence being overweight and obesity, prevention and treatment also require a multifactorial approach. One of the main strategies used to reduce this prevalence is the promotion of healthy habits, mainly through diet and exercise plans, which can be reinforced with psychological therapy and behavior change strategies [3].

Over recent years, the health sector has witnessed the development and expansion of health-related mobile apps [4]. It has been estimated that over 325,000 health apps were on the market in 2017 [5]. Such ubiquity can lead to an uncritical, implicit trust in apps—*apptimism* [6]—however, the majority of these apps are rarely downloaded, and their efficacy is not always evident [7]. There has been mixed evidence on whether mHealth apps improve long-term health and well-being [4]. While some apps have demonstrated efficacy in definitive trials, others have performed poorly [8].

A number of studies [9-14] have attempted to identify why health apps are not attaining their goal. Poor quality, a lack of guidance on an app’s usefulness, and low levels of support or lack of engagement from health professionals appear to be the most significant factors [9]. The assessment, validation, evaluation, and certification of health apps is a controversial topic among various stakeholders, and some guidelines and frameworks have already been published [10-14]; however, there is a lack of specific instruments to accurately assess health-related apps.

In recent years, there has been a rise in the use of apps intended to prevent weight problems or treat adults who are overweight or obese; these apps facilitate the tracking of physical and dietary patterns, provide recommendations and advice, or include motivational strategies to achieve personalized goals; however, evidence supporting the criteria used to assess the efficacy of these apps is scarce [3,4,15].

The main objective of the EVALAPPS project is to design and validate an instrument to assess the efficacy, safety, and potential effectiveness of health-related apps that are intended to manage weight and prevent obesity. A systematic review [16] to identify efficacy criteria used in previous validation studies has been carried out and a set of criteria were identified. These criteria, together with those defined by recently published frameworks for mHealth assessment, require validation and prioritization in order for the EVALAPPS instrument to be designed.

This paper focuses on the process of reaching a consensus among a broad group of stakeholders for a comprehensive set of criteria to be used in the EVALAPPS assessment instrument. First, the robustness of criteria that had been identified through literature review was validated. Second, criteria were prioritized using a quantitative approach based on the importance decided by stakeholders and the level of consensus among the stakeholders.

## Methods

### Study Design

**Figure 1:**
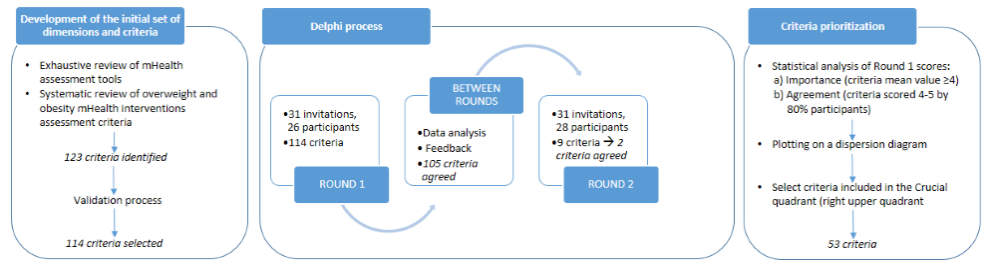
Methodological workflow.

A modified Delphi process was used with the domains and criteria that had been identified in a systematic review [16] in order to reach a consensus on criteria to be included in the EVALAPPS assessment instrument. The Delphi process is used to achieve expert consensus on a specific theme by voting and providing feedback through several consultation rounds [17]. In this study, the modified Delphi process consisted of an asynchronous online version of the original process. This modification provided the opportunity both to include experts from various geographical locations and to avoid the possibility of the influence of reputation or personality on the discussion. The methodological workflow is shown in Figure 1.

### Participant Recruitment

The scientific committee of the EVALAPPS project conducted a snowball sampling procedure to identify potential participants for the modified Delphi process. Snowball sampling is a nonprobability sampling technique in which existing participants recruit from their own contacts [18]. This sampling method is used in qualitative studies intended to reach a consensus and when statistical significance is not required. The initial sample for this study was selected by the research team. A total of 31 participants were selected using their profiles to ensure a representative panel of experts according to areas of expertise (physicians, health professionals, health managers and planners, technical experts such as developers, information and communication technology managers and digital entrepreneurs, and patients) and to ensure gender and geographic diversity. These experts were invited to participate in the modified Delphi process via email. Those who accepted were sent an email with a link to the round 1 survey.

### Initial Dimensions and Criteria

Development of the initial set of dimensions and criteria was based on (1) a review of criteria used by several mHealth assessment tools that was conducted through database searches—PubMed, PsycINFO, Scopus, Trip Medical Database, Clinical Trials Register, and Cochrane—and complemented with a manual search (up to May 2018) (Multimedia Appendix 1) and (2) evidence gathered by the EVALAPPS team through a systematic review [16] to identify efficacy, safety, and potential effectiveness criteria used to assess weight, overweight, and obesity management in mHealth interventions.

A total of 123 criteria were identified and classified according to purpose of the app (monitoring, treatment, or guideline), safety and privacy, clinical effectiveness, reliability (quality of contents), usability, functionality (browsing), level of development (interoperability), and 3 health indicators—personal data, physical state data, and activity data (Multimedia Appendix 2).

Based on the 123 criteria and 10 dimensions, a pilot survey was designed by a researcher unconnected with the EVALAPPS project. This survey was verified through an iterative (2 rounds) internal validation process by the research team, who reviewed the criteria and proposed corrections and clarifications. After the internal validation process, 114 criteria were selected for the Delphi process—3 in purpose of the app, 24 in safety and privacy, 11 in clinical effectiveness, 8 in reliability, 17 in usability, 9 in functionality, 5 in level of development, 6 in personal data, 15 in physical state data, and 16 in activity data (Multimedia Appendix 3).

### Round 1

The first round of the modified Delphi took place from April 14, 2018 to April 21, 2018. The 31 experts that had been selected received an email with information about the project, the objectives of the modified Delphi, and a link to the survey.

**Figure 2:**
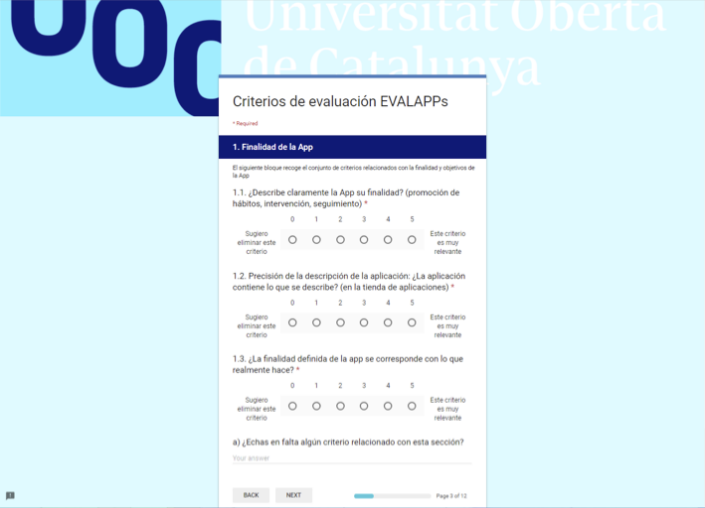
Screen capture of the online survey for round 1.

The survey was created using Google Forms and contained (1) a questionnaire to gather sociodemographic data about participants (age, gender, professional profile, and degree of expertise in mHealth) and (2) the set of criteria. Criteria were presented clustered by dimensions (Figure 2). Each criterion was polled according to its relevance using a 6-point Likert scale with extremes labeled (0, “I suggest this criterion is excluded”; 5, “This criterion is extremely relevant”). The Google Form included an open-ended blank space at the end of each dimension in which experts could post comments, provide additional information (eg, propose new criteria), and make clarifications. A reminder was sent 2 days before the deadline to those who had not completed the survey.

### Between Rounds

Data obtained in round 1 were collected and analyzed. The participation percentage was calculated and a descriptive analysis of the sample of experts was performed. For each criterion, the mean, standard deviation, median, interquartile range, and relative interquartile range of the ratings were calculated. SPSS software (version 21.0; IBM Corp) was used for statistical analysis. A criterion was accepted if the median sample rating was 4 or higher and the relative interquartile range was 0.67 or lower. Summaries of open-ended answers, including suggestions for changes or additional criteria, were presented to the research team before round 2.

The criteria that did not reach the set agreement level were included in a second round of the modified Delphi process. A new survey was designed with the objective of determining the validity of these criteria. Results obtained in round 1 were shared with experts via email, along with a link to the round 2 survey.

### Round 2

**Figure 3:**
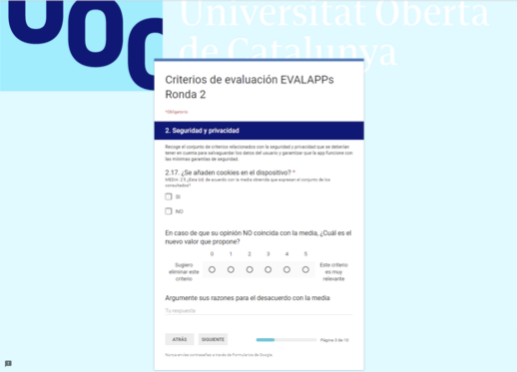
Screen capture of the online survey for round 2.

Round 2 of the modified Delphi was carried out from May 5, 2018 to May 14, 2018. The survey was created using Google Forms and was distributed to the same experts (n=31) (Figure 3).

The criteria that were discarded in round 1 were presented by dimension. Participants were asked whether they agreed or disagreed with the mean value obtained in round 1 for those criteria. If they disagreed, they were requested to provide a value for the criterion (using the same 6-point Likert scale as in round 1) and their reasoning (open-ended answer). No sociodemographic data were collected in this survey. A gentle reminder was sent 2 days before the deadline to those who had not completed the survey.

For each criterion, the mean, standard deviation, median, interquartile range, relative interquartile range, and also the relative frequency (number of responses that agreed with the round 1 mean/total number of responses) of the ratings were calculated for round 2. A criterion was accepted if the relative frequency of response was equal or greater than 80%. The research team then reviewed all open-ended answers.

### Prioritization

The scores obtained in round 1 were used to prioritize the criteria. Crucial criteria were defined in accordance with Ruiz Olabuénaga et al [19]. Criteria were plotted on a dispersion diagram by (1) the importance and (2) by the level of consensus and were classified as crucial, critical, circumstantial, marginal, or irrelevant according to their position with respect to quadrants defined by importance and level of consensus thresholds, respectively: (1) a mean rating of 4.00 or higher and (2) a level of consensus of at least 80% (ie, at least 80% rated the criteria as either 4 or 5).

### Ethics

The EVALAPPS project was approved by the ethics committee of the Universitat Oberta de Catalunya. An informed consent statement including a brief description of the project and the conditions of participation (voluntary participation, confidentiality, and data privacy) was provided for participants to sign prior to participation in each round.

## Results

### Expert Panel

Of the 31 experts invited to participate, 26 accepted (83.9% response rate) in round 1 and 28 accepted (90.3% response rate) in round 2. Round 1 respondents ranged in age from 31 to 70 years; 66.7% (17/26) were men and 33.3% (9/26) were women. Most participants were clinicians (16/26, 61.5%), followed by researchers (3/26, 11.5%). Most respondents in round 1 (17/26, 65.3%) identified themselves as expert or very expert (4 or 5) with respect to mHealth apps.

**Table 1:** Expert panel round 1.

Characteristics		Respondents (n=26), n (%)
**Gender**		
	Male	17 (66.7)
	Female	9 (33.3)
**Age**		
	<20	0 (0.0)
	20-30	0 (0.0)
	31-40	7 (26.9)
	41-50	8 (30.8)
	51-60	7 (26.9)
	61-70	4 (15.4)
	>70	0 (0.0)
**Respondent profile**		
	Clinicians	16 (61.5)
	University or research centers	3 (11.5)
	Technology-related position	2 (7.7)
	Consultant	2 (7.7)
	Other^a^	3 (11.5)
**Self-reported knowledge of health apps**		
	1 (low)	1 (3.8)
	2	3 (11.5)
	3	5 (19.2)
	4	14 (53.8)
	5 (very expert)	3 (11.5)

^a^Included 1 expert from an insurance enterprise, 1 person working in a government institution, and 1 retired civil servant.

### Round 1

Round 1 voting included 114 potential criteria of which 105 (92.1%) were deemed sufficiently relevant to be included (according to the inclusion thresholds) and 9 (7.9%) whose relevance were considered doubtful. Table 2 shows which criteria did not meet the importance or level of consensus inclusion thresholds during round 1. Respondents (11/26, 42.3%) provided additional comments, observations, and clarifications to the criteria during the round 1. In addition to the open-ended answers, 6 respondents (19%) also suggested additional criteria. The proposed criteria included use of inclusive language, information about risk factors, information about emotional well-being, ability to record diet type (vegan, Mediterranean, etc), functionality to export data in an open format, and distinction between use for kids or adolescents and adults.

**Table 2:** Criteria whose ratings did not reach the inclusion thresholds in round 1.

Dimension and criteria		Mean (SD)	Median (IQR)	Relative IQR
**Security and privacy**				
	Are cookies added to the device?	2.5 (1.45)	3 (2)	0.67
**Clinical effectiveness**				
	Does the app appear valued with at least a reasonable value in the app store, website, etc)?	3.2 (1.02)	3 (1)	0.33
	Does the app avoid the use of logos or other elements that may lead to a conflict of interest?	3.0 (1.46)	3 (2)	0.67
**Usability**				
	Does the app provide information about long-term use?	3.3 (1.08)	3 (1)	0.33
	Does the app inform users about possible malfunctions?	3.4 (1.17)	3 (1)	0.33
	Does the app include use options for left-handed people?	2.8 (1.43)	3 (2)	0.67
**Functionality**				
	Does the app always need to use an active internet connection?	2.5 (1.33)	3 (2)	0.67
**Personal data^a^**				
	Does the app contain options to record the user's family health history?	3.3 (1.35)	3 (2)	0.67
**Physical state data^a^**				
	In the event that the app contains options to record the height of the user, can it be done progressively over time?	2.8 (1.77)	3 (3)	1.00
	Does the app contain options to record the user’s resting pulse?	3.3 (1.57)	3.5 (3)	0.86
**Activity data^a^**				
	Does the app contain options to record the user's consumption of other toxins?	3.3 (1.65)	4 (3)	0.75
	Does the app contain options for recording the quality of the user's sleep?	3.2 (1.26)	3 (2)	0.67

^a^These dimensions are health indicators.

### Round 2

Participants were asked to rerate the 9 criteria whose initial ratings did not reach the inclusion thresholds in round 1 (Table 2). Of the 7 criteria, 2 were included as a result of round 2—“Does the app contain options for recording the quality of the user's sleep?” and “Does the app contain options to register substance abuse?”; the others were rejected with each receiving an opinion from at least one expert about why it should be rejected. A third round of voting was not required.

### Priority Criteria

A total of 63 criteria were obtained based on importance (mean rating≥4.00), and a total of 56 criteria were obtained based on level of consensus (at least 80% of ratings≥4). Using either prioritization strategy, these criteria included 14 in the safety and privacy dimension (importance: 14/63, 22.2%; level of consensus: 14/56, 25.0%) and 9 in the usability dimension (importance: 9/63, 14.3.2%; level of consensus: 9/56, 16.0%). When using importance, criteria in activity data (7/63, 11.1%), physical state data (7/63, 11.1%), and reliability (7/63, 11.1%) dimensions were also prioritized, and when using level of consensus, clinical effectiveness (6/56, 10.7%) was prioritized (Table 3).

The top 10 criteria are presented prioritized according to importance (Table 4) and level of consensus (Table 5). Two criteria were found to be high priorities using both methods of prioritization: “Are health recommendations offered by the app based on data collected in accordance with scientific evidence?” (importance: mean 4.77, SD 0.51; level of consensus: 25/26, 96.2%) and “Does the app correctly manage access to personal information through prior approval by the user?” (importance: mean 4.62, SD 0.50; level of consensus: 26/26, 100%). “At the request of the owner, can the supplier delete the app and any related data in the tracking system and documentation of access to the data to avoid any unauthorized access to personal data?” was the only other criteria to have unanimous consensus (26/26, 100%). The dimension with the most criteria prioritized in the top 10 was safety and privacy (importance: 3 criteria; level of consensus: 4 criteria).

**Figure 4:**
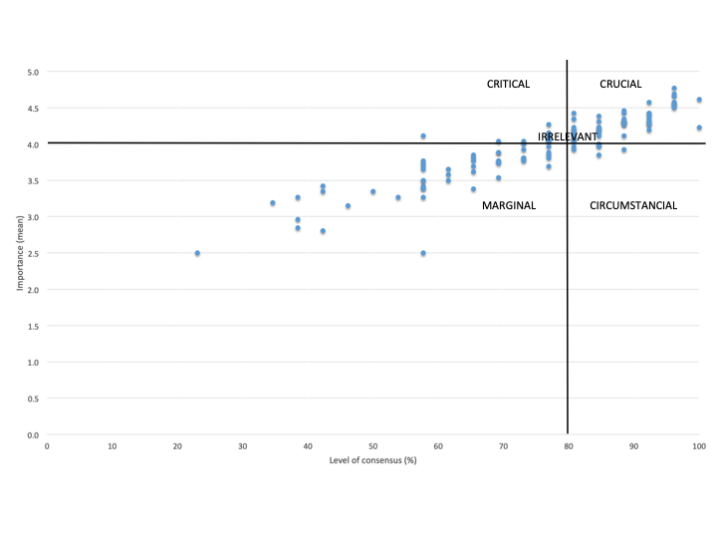
Criteria categorization according to their importance and level of consensus.

When criteria were plotted on a dispersion diagram (Figure 4), there were 53 criteria classified as crucial (Multimedia Appendix 3), mostly from the safety and privacy (13/53, 24.5%) and usability (9/53, 17.0%) dimensions. Dimensions maintained the same proportion of criteria after prioritization (Table 3).

**Table 3:** Number and percentage of criteria (by dimension) that were prioritized with each strategy.

Dimensions		Criteria, n (%)	Importance, n (%)	Consensus, n (%)	Crucial, n (%)
**All**		114 (100)	63 (100)	56 (100)	53 (100)
	Purpose of the app	3 (2.6)	3 (4.8)	3 (5.4)	3 (5.7)
	Safety and privacy	24 (21.1)	14 (22.2)	14 (25.0)	13 (24.5)
	Clinical effectiveness	11 (9.6)	5 (7.9)	6 (10.7)	5 (9.4)
	Reliability	8 (7.0)	7 (11.1)	5 (8.9)	5 (9.4)
	Usability	17 (14.9)	9 (14.3)	9 (16.0)	9 (17.0)
	Functionality	9 (7.9)	3 (4.8)	3 (5.4)	3 (5.7)
	Level of development	5 (4.4)	4 (6.3)	4 (7.1)	4 (7.5)
	Health indicator: personal data	6 (5.3)	4 (6.3)	2 (3.6)	2 (3.8)
	Health indicator: physical state data	15 (13.2)	7 (11.1)	4 (7.1)	4 (7.5)
	Health indicator: activity data	16 (14.0)	7 (11.1)	6 (10.7)	5 (9.4)

**Table 4:** The top 10 criteria according to importance.

Criteria	Dimension	Rating, mean (SD)
Are health recommendations offered by the app based on data collected in accordance with scientific evidence?	Clinical effectiveness	4.77 (0.51)
Is the app available on iOS and Android?	Level of development	4.69 (0.68)
Does the defined purpose of the app correspond to what it actually does?	Purpose of the app	4.65 (0.56)
Does the app correctly manage access to personal information through prior approval by the user?	Safety and privacy	4.62 (0.50)
Does the app clearly describe its purpose?	Purpose of the app	4.58 (0.58)
Does the app have a friendly and intuitive interface?	Usability	4.58 (0.58)
Is the app easy to use?	Functionality	4.58 (0.58)
Does the app have a privacy policy?	Safety and privacy	4.54 (0.58)
Is the content of the app correct, well written and relevant to the objective?	Reliability	4.54 (0.58)
Can the user choose not to participate in data transfer?	Safety and privacy	4.50 (1.03)

**Table 5:** The top 10 criteria according to level of consensus.

Criteria	Dimension	Ratings≥4 (n=26), n (%)
Does the app correctly manage access to personal information through prior approval by the user?	Safety and privacy	(26) 100
At the request of the owner, can the supplier delete the app and any related data in the tracking system and documentation of access to the data to avoid any unauthorized access to personal data?	Safety and privacy	(26) 100
Does the defined purpose of the app correspond to what it actually does?	Purpose of the app	(25) 96.2
Does the app have a privacy policy?	Safety and privacy	(25) 96.2
Can the user choose not to participate in data transfer?	Safety and privacy	(25) 96.2
Are health recommendations offered by the app based on data collected in accordance with scientific evidence?	Clinical effectiveness	(25) 96.2
Is the content of the app correct, well written and relevant to the objective?	Reliability	(25) 96.2
Does the app have a friendly and intuitive interface?	Usability	(25) 96.2
Is the app easy to use?	Functionality	(25) 96.2
Is the app available on iOS and Android?	Level of development	(25) 96.2

## Discussion

### Principal Findings

The EVALAPPS project was established to design and validate an instrument for assessing the efficacy, security, and potential effectiveness of mHealth apps intended to prevent and manage overweight conditions and obesity. The aim of this paper was to achieve consensus among a broad group of Spanish stakeholders on a comprehensive set of criteria, which will be used to guide development of the EVALAPPS assessment instrument. The criteria identified here were not only based on those found in literature, but have also been approved by major stakeholders, such as clinicians and app developers, using a modified Delphi process. The Delphi process was considered the most appropriate method for gathering information from experts in diverse locations in a valid and robust manner, while also avoiding individual influence and dominance. After 2 rounds and in combination with a statistical approach, the Delphi process both confirmed the robustness of the criteria that were identified (107/114, 94% of the criteria reached the consensus level) and generated a list of 53 criteria that were considered crucial for the EVALAPPS evaluation instrument (Multimedia Appendix 3). These criteria were largely from the security and privacy, usability, activity data (a health indicator), clinical effectiveness, and reliability dimensions.

### Comparison With Other Studies

There have been several initiatives that have evaluated or certified health apps, in general, and thematic apps, in particular (such as nutrition or mental health–related apps).

In 2016, the World Health Organization mHealth Technical Evidence Review Group published a mobile health evidence reporting and assessment checklist [20] which included 16 items: infrastructure, technology platform, interoperability, intervention delivery, intervention content, usability testing, user feedback, access of individual participants, cost assessment, adoption inputs, limitations for delivery at scale, contextual adaptability, replicability, data security, compliance with national guidelines or regulatory statutes, and fidelity of the intervention. The National Health System of the United Kingdom [21] has also recently published a set of criteria for health app assessment, grouped in the following domains: evidence of effectiveness, regulatory approval, clinical safety, privacy and confidentiality, security, usability and accessibility, interoperability, and technical stability. The Royal College of Physicians in the United Kingdom has published a checklist relating to health app assessment [22] that considers 3 main questions: “Who developed the app and what’s inside it?”; “How well does the app work?”; and “Is there any evidence that the app does actually alleviate the problem?” In 2015, Stoyanov et al [23] published the Mobile App Rating Scale for the evaluation of health apps in general. Mobile App Rating Scale includes 23 criteria grouped in several domains: app quality, engagement, functionality, aesthetics, information, and app subjective quality. The Mobile App Rating Scale also supplies optional items that can be modified to assess knowledge, attitudes, and intention to change, but these are not included in the main Mobile App Rating Scale scoring system.

There are other assessment initiatives focused on specific thematic apps. For instance, in 2017, DiFilippo et al [24] published the Nutrition App Quality Evaluation tool to evaluate nutrition-related apps. The Nutrition App Quality Evaluation includes a set of 25 items grouped in 5 blocks: app purpose, behavior change, knowledge and skill development, app functionality, adding information about the app, and the user. The Nutrition App Quality Evaluation includes 5 additional items to assess app appropriateness for different age groups and 4 additional items to assess apps for specific audiences. For mental health apps, the American Psychiatric Association [25] has proposed an app evaluation model based on 5 steps: gather information, risk/privacy and security, evidence, ease of use, and interoperability.

It is worth noting that the domains and criteria common to most of these initiatives largely relate to information about the app and the target users, content of the intervention, evidence, technological issues, privacy and security, interoperability and usability, and user experience–related issues. These criteria are aligned with domains proposed by international evaluation initiatives such as the European Network on Health Technology Assessment and its core model for the evaluation of health technology [26].

The criteria subjected to the Delphi process were those used in the initiatives and assessment models above, thus the final set of criteria for EVALAPPS aligns with current scholarship in the field. App quality, in terms of ensuring the security and privacy of user data (13/53, 24.5%) and usability (9/53, 17.0%), was considered of higher importance than clinical aspects, both in the literature review and by our professional panel. These results are surprising considering that clinicians formed the majority of the expert panel. Several authors have pointed out that assessing the clinical aspects of health apps is an immense challenge and at times this aspect is not considered or not sufficiently assessed [27]. This could be attributed to several factors, such as lack of a systematic assessment process for evaluating clinical aspects of health apps [27] and the fact that traditional methodologies for demonstrating clinical efficacy are inadequate in the constantly changing and evolving field of mHealth [28], in which technology and research develop independently [29]. Future challenges include the development of new assessment strategies for demonstrating clinical efficacy and achieving a balance in evaluation protocols between the potential effectiveness of health apps and technology-related aspects, such as usability and security.

Finally, this study revealed the relevance of a new dimension—health information collected by the app—which represented 20.7% of the final criteria set. To our knowledge, this is the first model to include criteria specifically for assessing the management of obesity and overweight conditions using mobile tools.

### Strengths and Limitations

Criteria were developed through a comprehensive review of evidence and consultation with a diverse expert panel using the Delphi process. A systematic literature review on domains and criteria to be included in the tool contributed to the study’s validity, as did the iterative study design and the pilot survey. In fact, the iterative process of this study is one of its main strengths. In addition, open-ended answers were included in each section, thereby enabling experts to provide comments on included criteria or provide new criteria for consideration. Validity was also enhanced by including several types of profiles on the expert panel. This ensured the criteria that were approved were relevant and generalizable.

The Delphi process itself has some limitations. Based on the participation of a small number of experts, the Delphi process can be affected by research questions and panel configuration [30,31]. Respondents from our panel included 26 experts, mainly with clinical profiles. Due to the backgrounds of the experts invited to participate, some criteria may not have been properly addressed, for instance, health professionals may not have expertise on technology privacy and security issues. The majority of experts were from Catalonia, one of the 17 regions of Spain. As the Spanish Health System is organized at the regional level with each autonomous community health system having its own priorities, this panel does not fully guarantee the external validity of results. Finally, this study did not include face-to-face meetings with respondents to discuss ratings, to investigate areas of disagreements, or to gain more in-depth insight.

### Future Actions and Conclusions

The findings of this study are important for both professionals and weight management–related technology users. The criteria agreed upon will be included in the EVALAPPS assessment instrument. Future measures include a cocreation session on the appearance and presentation of this instrument. This will be undertaken by the research team and technology experts, who will discuss such aspects as whether the tool should be in a web or app format and what components will be included. The instrument will then be piloted on various weight control apps. The findings of this study contribute to the literature on both mHealth evaluation and weight management apps.

## Appendix

Multimedia Appendix 1 mHealth assessment tools reviewed for the development of the initial set of dimensions and criteria.

Multimedia Appendix 2 Initial set of dimensions and criteria identified - pilot survey format.

Multimedia Appendix 3 List of criteria under consensus.

## Abbreviations

mHealth: mobile health
