# Correction: Evaluation Criteria for Weight Management Apps: Validation Using a Modified Delphi Process

**DOI:** 10.2196/47584

**Published:** 2023-04-10

**Authors:** Noemí Robles, Elisa Puigdomènech Puig, Corpus Gómez-Calderón, Francesc Saigí-Rubió, Guillem Cuatrecasas Cambra, Alberto Zamora, Montse Moharra, Guillermo Paluzié, Mariona Balfegó, Carme Carrion

**Affiliations:** 1 eHealth Lab Research Group Universitat Oberta de Catalunya Barcelona Spain; 2 eHealth Center Universitat Oberta de Catalunya Barcelona Spain; 3 Red de Investigación en Servicios de Salud en Enfermedades Crónicas Barcelona Spain; 4 Agència de Qualitat i Avaluació Sanitàries de Catalunya Barcelona Spain; 5 Marina Salud Alicante Spain; 6 Faculty of Health Sciences Universitat Oberta de Catalunya Barcelona Spain; 7 Interdisciplinary Research Group on ICTs Universitat Oberta de Catalunya Barcelona Spain; 8 Clínica Sagrada Família CPEN SL Servei d'Endocrinologia i Nutrició Barcelona Spain; 9 Corporació de Salut del Maresme i la Selva Hospital de Blanes Blanes Spain; 10 Grup de Medicina Traslacional i Ciències de la Decisió Universitat de Girona Girona Spain; 11 Centro de Investigación Biomédica en Red en Epidemiología y Salud Pública Barcelona Spain

In "Assessment of the Efficacy, Safety, and Effectiveness of Weight Control and Obesity Management Mobile Health Interventions: Systematic Review" (JMIR Mhealth Uhealth 2020;8(7):e16899) the authors noted one clarification that should be added:

The Acknowledgments section reads as:

All authors contributed equally. The research for this paper was fully funded by the Instituto de Salud Carlos III from the Spanish Ministry of Science, Innovation and Universities, grant number PI16/01764.

And will be changed to:

All authors contributed equally. The research for this paper was fully funded by the Instituto de Salud Carlos III from the Spanish Ministry of Science, Innovation and Universities, grant number PI16/01764 co-funded by FEDER.

The correction will appear in the online version of the paper on the JMIR Publications website on April 10, 2023 together with the publication of this correction notice. Because this was made after submission to PubMed, PubMed Central, and other full-text repositories, the corrected article has also been resubmitted to those repositories.

