# Supplementary material for: Evaluation Criteria for Weight Management Apps: Validation Using a Modified Delphi Process
Source: JMIR Mhealth Uhealth. 2020 Jul 22;8(7):e16899. doi: 10.2196/16899 (PMC7407251; doi:10.2196/16899)
Supplement: Multimedia Appendix 1 [file mhealth_v8i7e16899_app1.pdf]

MULTIMEDIA APPENDIX 1
mHealth assessment tools reviewed for the development of the initial set of dimensions and criteria

Agarwal S, LeFevre A, Lee J, L'Engle K, Mehl G, Sinha C, et al. A Guidelines for reporting of health interventions using mobile phones: mobile health (mHealth) evidence reporting and assessment (mERA) checklist. BMJ 2016; 352

Stoyanov SR, Hides L, Kavanagh DJ, Zelenko O, Tjondronegoro D, Mani M. Mobile App Rating Scale: A New Tool for Assessing the Quality of Health Mobile Apps. JMIR Mhealth Uhealth 2015;3(1):e27Stoyanov et al. 2015

Wyatt JC, Thimbleby H, Rastall P, Hoogewerf J, Wooldridge D, Williams J. What makes a good clinical app? Introducing the RCP Health Informatics Unit checklist. Clin Med J R Coll Physicians London 2015; [doi: 10.7861/clinmedicine.15-6-519]

DiFilippo KN, Huang W, Chapman-Novakofski KM. A New Tool for Nutrition App Quality Evaluation (AQEL): Development, Validation, and Reliability Testing. JMIR mHealth uHealth 2017; [doi: 10.2196/mhealth.7441]

Public Health England. Guidance. Criteria for health app assessment [Internet]. 2017. Available from: https://www.gov.uk/government/publications/health-app-assessment-criteria/criteria-for-health-app-assessment

American Psychiatric Association. Mental Helath Apps [Internet]. Available from: https://www.psychiatry.org/psychiatrists/practice/mental-health-apps

European Network for Health Technology Assessment (EUnetHTA). HTA Core Model [Internet]. Available from: https://www.eunethta.eu/hta-core-model/

Myhealthapps:http://myhealthapps.net/

Fundacion iSYS (Internet, Salud y Sociedad): fundacionisys.org

ORCHA: https://www.orcha.co.uk/

iMedicalApps: www.imedicalapps.com

Happtique: http://www.happtique.com/

Distintivo App Saludable: www.calidadappsalud.com/distintivo-appsaludable

Acreditación ‘Appsalut’: http://www.ticsalut.cat/mhealth/portal-appsalut/acreditacio/

Apps library NHS: https://www.nhs.uk/apps-library/
