# Supplementary material for: Evaluation Criteria for Weight Management Apps: Validation Using a Modified Delphi Process
Source: JMIR Mhealth Uhealth. 2020 Jul 22;8(7):e16899. doi: 10.2196/16899 (PMC7407251; doi:10.2196/16899)
Supplement: Multimedia Appendix 2 [file mhealth_v8i7e16899_app2.pdf]

Criterios de evaluación EVALAPPs

Le agradeceríamos su colaboración para responder este cuestionario que forma parte de la investigación que se está llevando a cabo dentro del proyecto EVALAPPS sobre la determinación de qué criterios deberían tenerse en cuenta a la hora de evaluar aplicaciones móviles en el ámbito de la gestión y prevención del sobrepeso y la obesidad.

Las listas de verificación tienen como objetivo comprobar la idoneidad de cumplimiento o no de determinados criterios.

La estructura propuesta para la obtención de consenso respecto a los criterios mas adecuados para evaluación de este tipo de dispositivos es la siguiente:

1. Finalidad de la app (monitorizar, guía, tratamiento)
2. Seguridad y privacidad
3. Efectividad clínica (evidencia)
4. Cantidad de información recibida o solicitada
5. Calidad de la información clínica (fiabilidad, credibilidad)
6. Usabilidad (adherencia, compromiso, entretenimiento, personalización, interactividad, experiencia del usuario)
7. Funcionalidades (facilidad de uso, navegación)
8. Estética (gráficos, diseño, atractiva)
9. Nivel de desarrollo (interoperabilidad)

Con este cuestionario se pretende:

- Identificar qué criterios deberían tenerse en cuenta en la evaluación de aplicaciones móviles en el ámbito del sobrepeso y la obesidad.

- Valorar la importancia de los distintos criterios de evaluación dentro de cada uno de los apartados.

- Eliminar aquellos criterios que no sean necesarios incluir en cada uno de los apartados.

- Añadir nuevos criterios que no estén incluidos en el cuestionario.

Responder al cuestionario le supondrá aproximadamente 10-15 minutos de su tiempo.

MUCHAS GRACIAS POR SU COLABORACIÓN

Equipo de trabajo de EVALAPPS.

Nota sobre la privacidad

Los registros de sus respuestas a la encuesta no contienen ninguna identificación suya a menos que una pregunta específicamente así lo haga. Si responde esta encuesta utilizando una contraseña que le da acceso al cuestionario, le garantizamos que esta no se asocia a ninguna de sus respuestas.

Los datos personales que se extraigan de esta encuesta serán confidenciales, serán tratados de acuerdo a la normativa de protección de datos de carácter personal vigente y sólo se utilizarán de forma agregada como base estadística del informe final y otras publicaciones científicas que se elaborarán.

*Obligatorio

1. **Dirección de correo electrónico** *

____

Proyecto financiado por el Instituto de Salud Carlos III, Ministerio Español de Economía y Competititividad, PI16/01764

Caracterización personal

2. **1. Nombre y apellidos** *

Los registros del cuestionario serán confidenciales. Se requiere su identificación con el fin de asociar la estadística de respuestas a las características personales y profesionales de los expertos.

____

3. **2. ¿Cuál es su género?** *

Marca solo un óvalo.

- ( ) Hombre
- ( ) Mujer
- ( ) Prefiero no responder

4. **3. ¿Cuál es su edad?** *

Marca solo un óvalo.

- ( ) < 20
- ( ) 20-30
- ( ) 31-40
- ( ) 41-50
- ( ) 51-60
- ( ) 61-70
- ( ) >70

5. **4. Centro de trabajo** *

Marca solo un óvalo.

- ( ) Universidad, Centro Universitario
- ( ) Institución gubernamental
- ( ) Empresa tecnológica
- ( ) Centro hospitalario o institución asistencial
- ( ) Centro de investigación
- ( ) Otro: ______________________________

6. **5. ¿Cómo se auto definiría en el conocimiento de las aplicaciones móviles (Apps) de salud?** *

Marca solo un óvalo.

	1	2	3	4	5	
Poco conocimiento	⬭	⬭	⬭	⬭	⬭	Soy experto

1. Finalidad de la App

El siguiente bloque recoge el conjunto de criterios relacionados con la finalidad y objetivos de la App

7. **6. ¿Describe claramente la App su finalidad?(promoción de hábitos, intervención, seguimiento)** *

Marca solo un óvalo.

	0	1	2	3	4	5	
Sugiero eliminar este criterio	⬭	⬭	⬭	⬭	⬭	⬭	Este criterio es muy relevante

8. **7. Precisión de la descripción de la aplicación: ¿La aplicación contiene lo que se describe? (en la tienda de aplicaciones)** *

Marca solo un óvalo.

	0	1	2	3	4	5	
Sugiero eliminar este criterio	⬭	⬭	⬭	⬭	⬭	⬭	Este criterio es muy relevante

9. **8. ¿Se corresponde lo que hace realmente la App con la finalidad que define?** *

Marca solo un óvalo.

	0	1	2	3	4	5	
Sugiero eliminar este criterio	⬭	⬭	⬭	⬭	⬭	⬭	Este criterio es muy relevante

10. **a) ¿Echas en falta algún criterio?**

2. Seguridad y privacidad

Recoge el conjunto de criterios relacionados con la seguridad y privacidad que se deberían tener en cuenta para salvaguardar los datos del usuario y garantizar que la app funcione con las mínimas garantías de seguridad.

11. **9. ¿Identifica la App los riesgos que su manejo puede suponer para la seguridad del usuario?** *

Marca solo un óvalo.

	0	1	2	3	4	5	
Sugiero eliminar este criterio	◯	◯	◯	◯	◯	◯	Este criterio es muy relevante

12. **10. ¿Gestiona la App correctamente el acceso a la información personal mediante la aprobación previa por parte del usuario?** *

Marca solo un óvalo.

	0	1	2	3	4	5	
Sugiero eliminar este criterio	◯	◯	◯	◯	◯	◯	Este criterio es muy relevante

13. **11. ¿La App tiene en cuenta las configuraciones seguras de redes para prevenir la interceptación de datos, con medidas de salvaguarda de seguridad específicas para redes móviles?.** *

Marca solo un óvalo.

	0	1	2	3	4	5	
Sugiero eliminar este criterio	◯	◯	◯	◯	◯	◯	Este criterio es muy relevante

14. **12. En el caso de que la App use servicios en la nube, ¿ declara los términos y condiciones de uso y garantiza su seguridad?** *

Marca solo un óvalo.

	0	1	2	3	4	5	
Sugiero eliminar este criterio	◯	◯	◯	◯	◯	◯	Este criterio es muy relevante

15. **13. Ante una pérdida o robo del dispositivo, ¿el usuario puede informar y bloquear el acceso al dispositivo?** *

Marca solo un óvalo.

	0	1	2	3	4	5	
Sugiero eliminar este criterio	◯	◯	◯	◯	◯	◯	Este criterio es muy relevante

16. **14. Los intentos de acceso no autorizados ¿pueden registrarse e informarse de inmediato al controlador de los datos?.** *

Marca solo un óvalo.

	0	1	2	3	4	5	
Sugiero eliminar este criterio	◯	◯	◯	◯	◯	◯	Este criterio es muy relevante

17. **15. A petición del propietario, ¿el proveedor puede eliminar la App y cualquier dato relacionado en el sistema de rastreo y documentación de acceso a los datos para evitar cualquier acceso no autorizado a datos personales?.** *

Marca solo un óvalo.

	0	1	2	3	4	5	
Sugiero eliminar este criterio	⬭	⬭	⬭	⬭	⬭	⬭	Este criterio es muy relevante

18. **16. ¿Incorpora la App avisos de privacidad y opciones para su configuración?.** *

Marca solo un óvalo.

	0	1	2	3	4	5	
Sugiero eliminar este criterio	⬭	⬭	⬭	⬭	⬭	⬭	Este criterio es muy relevante

19. **17. ¿Recoge la App únicamente los datos imprescindibles (según las normas médicas) y la información esencial para su funcionamiento?.** *

Marca solo un óvalo.

	0	1	2	3	4	5	
Sugiero eliminar este criterio	⬭	⬭	⬭	⬭	⬭	⬭	Este criterio es muy relevante

20. **18. ¿La App permite identificar claramente a las personas que realizan acciones (cambios, eliminaciones y/o incorporaciones) en las bases de datos?.** *

Marca solo un óvalo.

	0	1	2	3	4	5	
Sugiero eliminar este criterio	⬭	⬭	⬭	⬭	⬭	⬭	Este criterio es muy relevante

21. **19. ¿La App informa a los usuarios, de forma clara y sencilla, respecto a la normativa legal sobre protección de datos personales?** *

Marca solo un óvalo.

	0	1	2	3	4	5	
Sugiero eliminar este criterio	⬭	⬭	⬭	⬭	⬭	⬭	Este criterio es muy relevante

22. **20. ¿La App informa a los usuarios , de forma clara y sencilla, sobre la facilitación de datos a terceros?** *

Marca solo un óvalo.

	0	1	2	3	4	5	
Sugiero eliminar este criterio	⬭	⬭	⬭	⬭	⬭	⬭	Este criterio es muy relevante

23. **21. ¿Contempla la App una política de privacidad?** *
Marca solo un óvalo.

	0	1	2	3	4	5	
Sugiero eliminar este criterio	⬭	⬭	⬭	⬭	⬭	⬭	Este criterio es muy relevante

24. **22. ¿Los datos personales del usuario son anonimizados?** *
Marca solo un óvalo.

	0	1	2	3	4	5	
Sugiero eliminar este criterio	⬭	⬭	⬭	⬭	⬭	⬭	Este criterio es muy relevante

25. **23. ¿El usuario puede optar por no participar en la cesión de datos?** *
Marca solo un óvalo.

	0	1	2	3	4	5	
Sugiero eliminar este criterio	⬭	⬭	⬭	⬭	⬭	⬭	Este criterio es muy relevante

26. **24. ¿El usuario puede borrar sus datos?** *
Marca solo un óvalo.

	0	1	2	3	4	5	
Sugiero eliminar este criterio	⬭	⬭	⬭	⬭	⬭	⬭	Este criterio es muy relevante

27. **25. ¿Se añaden cookies en el dispositivo?** *
Marca solo un óvalo.

	0	1	2	3	4	5	
Sugiero eliminar este criterio	⬭	⬭	⬭	⬭	⬭	⬭	Este criterio es muy relevante

28. **26. ¿Informa la App sobre qué datos se comparten?** *
Marca solo un óvalo.

	0	1	2	3	4	5	
Sugiero eliminar este criterio	⬭	⬭	⬭	⬭	⬭	⬭	Este criterio es muy relevante

29. **27. ¿Informa la App sobre con quien se comparten los datos?** *
Marca solo un óvalo.

	0	1	2	3	4	5	
Sugiero eliminar este criterio	⬭	⬭	⬭	⬭	⬭	⬭	Este criterio es muy relevante

30. **28. ¿Informa la App sobre el almacenamiento de los datos en el dispositivo o en la web (es decir, "la nube") o en ambos?** *

Marca solo un óvalo.

	0	1	2	3	4	5	
Sugiero eliminar este criterio	◯	◯	◯	◯	◯	◯	Este criterio es muy relevante

31. **29. ¿Informa la App sobre qué medidas de seguridad existen?** *

Marca solo un óvalo.

	0	1	2	3	4	5	
Sugiero eliminar este criterio	◯	◯	◯	◯	◯	◯	Este criterio es muy relevante

32. **30. ¿Los datos que almacena la App están encriptados en el dispositivo y el servidor?** *

Marca solo un óvalo.

	0	1	2	3	4	5	
Sugiero eliminar este criterio	◯	◯	◯	◯	◯	◯	Este criterio es muy relevante

33. **31. ¿Expresa claramente la App su adecuación a la normativa vigente en materia de privacidad y seguridad?** *

Marca solo un óvalo.

	0	1	2	3	4	5	
Sugiero eliminar este criterio	◯	◯	◯	◯	◯	◯	Este criterio es muy relevante

34. **32. ¿Contempla la App aspectos de seguridad relativos a efectos adversos?** *

Marca solo un óvalo.

	0	1	2	3	4	5	
Sugiero eliminar este criterio	◯	◯	◯	◯	◯	◯	Este criterio es muy relevante

35. **a) ¿Echas en falta algún criterio respecto a Seguridad/Privacidad ?**

____

____

____

____

____

3. Efectividad clínica

El siguiente bloque recoge el conjunto de criterios relacionados con la efectividad clínica (evidencia)

36. **33. ¿La aplicación tiene objetivos específicos adecuados, medibles y alcanzables (especificados en la descripción de la tienda de aplicaciones o dentro de la aplicación)?** *

Marca solo un óvalo.

	0	1	2	3	4	5	
Sugiero eliminar este criterio							Este criterio es muy relevante

37. **34. ¿Hay evidencia publicada y revisada por pares sobre la herramienta?** *

Marca solo un óvalo.

	0	1	2	3	4	5	
Sugiero eliminar este criterio							Este criterio es muy relevante

38. **35. ¿La App cita y prioriza fuentes fiables de información con evidencia científica (rigor y calidad, avales científicos…)?** *

Marca solo un óvalo.

	0	1	2	3	4	5	
Sugiero eliminar este criterio							Este criterio es muy relevante

39. **36. Si las fuentes de información no son reconocidas científicamente, ¿se indica cómo se elaboró la información y que relevancia y fiabilidad tienen las fuentes?.** *

Marca solo un óvalo.

	0	1	2	3	4	5	
Sugiero eliminar este criterio							Este criterio es muy relevante

40. **37. ¿Constan las revisiones y actualizaciones de la App según nueva evidencia científica?.** *

Marca solo un óvalo.

	0	1	2	3	4	5	
Sugiero eliminar este criterio							Este criterio es muy relevante

41. **38. ¿La App aparece valorada con, al menos, un valor razonable en tienda de aplicaciones, sitio web, etc.)?** *

Marca solo un óvalo.

	0	1	2	3	4	5	
Sugiero eliminar este criterio							Este criterio es muy relevante

42. **39. ¿La App ofrece contenidos y servicios útiles y de valor de acuerdo con el público objetivo, adaptándose a los destinatarios a los que se dirige?.** *
Marca solo un óvalo.

	0	1	2	3	4	5	
Sugiero eliminar esta sugerencia	◯	◯	◯	◯	◯	◯	Esta sugerencia es muy relevante

43. **40. ¿La App emplea un lenguaje acorde a la audiencia?. (En términos generales, un lenguaje comprensible, llano y sencillo, con mensajes adaptados al perfil de usuario en cuanto a estilo y nivel lingüístico, facilitando la comprensión del usuario y evitando utilizar tecnicismos).** *
Marca solo un óvalo.

	0	1	2	3	4	5	
Sugiero eliminar esta sugerencia	◯	◯	◯	◯	◯	◯	Esta sugerencia es muy relevante

44. **41. ¿La App emplea sistemas de datos fiables y válidos, reconocidos por profesionales médicos, especialistas, sociedades científicas y/o organismo de salud?.** *
Marca solo un óvalo.

	0	1	2	3	4	5	
Sugiero eliminar esta sugerencia	◯	◯	◯	◯	◯	◯	Esta sugerencia es muy relevante

45. **42. Las recomendaciones de salud que hace la App en base a los datos recogidos ¿se adecúan a la evidencia científica?.** *
Marca solo un óvalo.

	0	1	2	3	4	5	
Sugiero eliminar esta sugerencia	◯	◯	◯	◯	◯	◯	Esta sugerencia es muy relevante

46. **43. ¿La App presenta estrategias para aumentar la adherencia del usuario? (como la gamificación, la capacidad de crear retos o recompensas, el registro de hábitos, etc.).** *
Marca solo un óvalo.

	0	1	2	3	4	5	
Sugiero eliminar esta sugerencia	◯	◯	◯	◯	◯	◯	Esta sugerencia es muy relevante

47. **44. ¿La App evita el uso de logos u otros elementos que puedan llevar a conflicto de interés?.** *
Marca solo un óvalo.

	0	1	2	3	4	5	
Sugiero eliminar esta sugerencia	◯	◯	◯	◯	◯	◯	Esta sugerencia es muy relevante

48. **a) ¿Echas en falta alguna Recomendación ?**

____

____

____

____

____

4. Cantidad de información

El siguiente bloque recoge el conjunto de criterios relacionados con la cantidad de la información recibida o solicitada

49. **45. La explicación visual de conceptos a través de tablas, gráficos, imágenes, videos, etc. ¿es suficiente, clara, lógica y correcta?** *

Marca solo un óvalo.

	0	1	2	3	4	5	
Sugiero eliminar este criterio	⬭	⬭	⬭	⬭	⬭	⬭	Este criterio es muy relevante

50. **a) ¿Echas en falta algún criterio?**

____

____

____

____

____

5. Calidad de la información clínica

El siguiente bloque recoge el conjunto de criterios relacionados con la calidad de la información clínica (fiabilidad, credibilidad)

51. **46. ¿El contenido de la aplicación es correcto, está bien escrito y es relevante para el objetivo?** *

Marca solo un óvalo.

	0	1	2	3	4	5	
Sugiero eliminar este criterio	⬭	⬭	⬭	⬭	⬭	⬭	Este criterio es muy relevante

52. **47. Credibilidad: ¿la aplicación proviene de una fuente legítima (especificada en la descripción de la tienda de aplicaciones o dentro de la propia aplicación)?** *

Marca solo un óvalo.

	0	1	2	3	4	5	
Sugiero eliminar este criterio	⬭	⬭	⬭	⬭	⬭	⬭	Este criterio es muy relevante

53. **48. La información clínica contenida en la App ¿cuenta con el aval de las sociedades científicas o colegios profesionales?** *
Marca solo un óvalo.

	0	1	2	3	4	5	
Sugiero eliminar este criterio	⬭	⬭	⬭	⬭	⬭	⬭	Este criterio es muy relevante

54. **49. La información clínica contenida en la App ¿cuenta con el aval de alguna organización de salud o autoridad sanitaria?** *
Marca solo un óvalo.

	0	1	2	3	4	5	
Sugiero eliminar este criterio	⬭	⬭	⬭	⬭	⬭	⬭	Este criterio es muy relevante

55. **50. ¿Advierte la App sobre conductas o propuestas inadecuadas? (Por ejemplo cuando hay riesgo de dietas muy restrictivas o cuando sea necesario consultar con un profesional)** *
Marca solo un óvalo.

	0	1	2	3	4	5	
Sugiero eliminar este criterio	⬭	⬭	⬭	⬭	⬭	⬭	Este criterio es muy relevante

56. **51. En el supuesto que la App venda productos o servicios, ¿proporciona información clara y comprensible de las condiciones del comercio electrónico?.** *
Marca solo un óvalo.

	0	1	2	3	4	5	
Sugiero eliminar este criterio	⬭	⬭	⬭	⬭	⬭	⬭	Este criterio es muy relevante

57. **52. En el supuesto que la App venda productos o servicios, ¿proporciona información clara sobre conflictos de intereses o declaraciones éticas al respecto?.** *
Marca solo un óvalo.

	0	1	2	3	4	5	
Sugiero eliminar este criterio	⬭	⬭	⬭	⬭	⬭	⬭	Este criterio es muy relevante

58. **53 ¿Hay constancia de que se ha testado la aplicación? debe ser verificado por evidencia (en la literatura científica publicada)** *
Marca solo un óvalo.

	0	1	2	3	4	5	
Sugiero eliminar este criterio	⬭	⬭	⬭	⬭	⬭	⬭	Este criterio es muy relevante

59. **a) ¿Echas en falta algún criterio?**

6. Usabilidad

El siguiente bloque recoge el conjunto de criterios relacionados con aquellos aspectos que se deberían tener en cuenta para que la interacción del usuario con la App sea fácil y permita alcanzar el objetivo establecido (adherencia, compromiso, entretenimiento, personalización, interactividad, experiencia del usuario)

60. **54. ¿Tiene la App una interfaz amigable e intuitiva?** *

Marca solo un óvalo.

	0	1	2	3	4	5	
Sugiero eliminar este criterio	⬭	⬭	⬭	⬭	⬭	⬭	Este criterio es muy relevante

61. **55. ¿La aplicación es divertida / entretenida de usar? ¿Utiliza la App alguna estrategia para aumentar la participación a través del entretenimiento (por ejemplo, a través de la gamificación)?** *

Marca solo un óvalo.

	0	1	2	3	4	5	
Sugiero eliminar este criterio	⬭	⬭	⬭	⬭	⬭	⬭	Este criterio es muy relevante

62. **56. ¿Las opciones de navegación y el contenido relevante son fácilmente percibidos por el usuario en un vistazo inicial?.** *

Marca solo un óvalo.

	0	1	2	3	4	5	
Sugiero eliminar este criterio	⬭	⬭	⬭	⬭	⬭	⬭	Este criterio es muy relevante

63. **57. ¿La navegación entre las secciones de la App es fácil, intuitiva y rápida?.** *

Marca solo un óvalo.

	0	1	2	3	4	5	
Sugiero eliminar este criterio	⬭	⬭	⬭	⬭	⬭	⬭	Este criterio es muy relevante

64. **58. ¿Presenta la App un diseño amigable en los formularios de introducción de datos?** *

Marca solo un óvalo.

	0	1	2	3	4	5	
Sugiero eliminar este criterio	⬭	⬭	⬭	⬭	⬭	⬭	Este criterio es muy relevante

65. **59. ¿La funcionalidad de cada elemento es claramente identificable? (por ejemplo, si el usuario debe hacer una acción concreta, la App debería indicarle clara y visualmente cuál es la acción que se debe hacer).** *

Marca solo un óvalo.

	0	1	2	3	4	5	
Sugiero eliminar este criterio	⬭	⬭	⬭	⬭	⬭	⬭	Este criterio es muy relevante

66. **60. ¿El funcionamiento de la App es rápido?** *

Marca solo un óvalo.

	0	1	2	3	4	5	
Sugiero eliminar este criterio	⬭	⬭	⬭	⬭	⬭	⬭	Este criterio es muy relevante

67. **61. ¿Avisa la App de las operaciones de larga duración?** *

Marca solo un óvalo.

	0	1	2	3	4	5	
Sugiero eliminar este criterio	⬭	⬭	⬭	⬭	⬭	⬭	Este criterio es muy relevante

68. **62. ¿Informa la App de los posibles fallos de funcionamiento?** *

Marca solo un óvalo.

	0	1	2	3	4	5	
Sugiero eliminar este criterio	⬭	⬭	⬭	⬭	⬭	⬭	Este criterio es muy relevante

69. **63. Personalización de la App: ¿Proporciona / retiene todas las configuraciones / preferencias necesarias para las características de las aplicaciones (por ejemplo, sonido, contenido, notificaciones, etc.)?** *

Marca solo un óvalo.

	0	1	2	3	4	5	
Sugiero eliminar este criterio	⬭	⬭	⬭	⬭	⬭	⬭	Este criterio es muy relevante

70. **64. Interactividad: ¿Permite la entrada del usuario, proporciona comentarios, contiene avisos (recordatorios, opciones de uso compartido, notificaciones, etc.)? Nota: estas funciones deben ser personalizables y no abrumadoras para ser perfectas.** *

Marca solo un óvalo.

	0	1	2	3	4	5	
Sugiero eliminar este criterio	⬭	⬭	⬭	⬭	⬭	⬭	Este criterio es muy relevante

71. **65. ¿El contenido de la aplicación (información visual, idioma, diseño) es apropiado para su público objetivo?** *
Marca solo un óvalo.

	0	1	2	3	4	5	
Sugiero eliminar este criterio	◯	◯	◯	◯	◯	◯	Este criterio es muy relevante

72. **66. ¿Utiliza la App recursos gráficos (como el contraste o la inversión de colores) para destacar qué elemento o apartado ha sido seleccionado y qué campos no han sido llenados de forma correcta o son incorrectos?** *
Marca solo un óvalo.

	0	1	2	3	4	5	
Sugiero eliminar este criterio	◯	◯	◯	◯	◯	◯	Este criterio es muy relevante

73. **67. En los cambios de registro de datos ¿introduce la App ayuda sobre qué información se necesita? (por ejemplo, descriptivos, desplegables relacionados, calendarios predeterminados para fechas, etc.).** *
Marca solo un óvalo.

	0	1	2	3	4	5	
Sugiero eliminar este criterio	◯	◯	◯	◯	◯	◯	Este criterio es muy relevante

74. **a) ¿Echas en falta algún criterio?**

____
____
____
____
____

7. Funcionalidades

El siguiente bloque recoge el conjunto de criterios relacionados con las funcionalidades, facilidad de uso y navegación

75. **68. ¿Es fácil aprender a usar la aplicación?** *
Marca solo un óvalo.

	0	1	2	3	4	5	
Sugiero eliminar este criterio	◯	◯	◯	◯	◯	◯	Este criterio es muy relevante

76. **69. Las funciones y componentes de la App (botones/menús) ¿funcionan con precisión y rapidez?** *
Marca solo un óvalo.

	0	1	2	3	4	5	
Sugiero eliminar este criterio							Este criterio es muy relevante

77. **70. ¿Presenta la App fallos de "congelación" que interrumpen su interacción? (por ejemplo, si el usuario acepta una llamada entrante mientras se ejecuta la App, debería ser posible retornar al mismo punto al final de la llamada).** *
Marca solo un óvalo.

	0	1	2	3	4	5	
Sugiero eliminar este criterio							Este criterio es muy relevante

78. **71. ¿Son claras las etiquetas de menú, los iconos y las instrucciones?** *
Marca solo un óvalo.

	0	1	2	3	4	5	
Sugiero eliminar este criterio							Este criterio es muy relevante

79. **72. Respecto a la navegación, ¿la App se mueve entre pantallas de un modo lógico, preciso, apropiado e ininterrumpido?** *
Marca solo un óvalo.

	0	1	2	3	4	5	
Sugiero eliminar este criterio							Este criterio es muy relevante

80. **73. ¿Todos los enlaces de pantalla necesarios están presentes?** *
Marca solo un óvalo.

	0	1	2	3	4	5	
Sugiero eliminar este criterio							Este criterio es muy relevante

81. **74. ¿Las interacciones de diseño gestual (toques, scrolls,...) son consistentes e intuitivas en todos los componentes y pantallas?** *
Marca solo un óvalo.

	0	1	2	3	4	5	
Sugiero eliminar este criterio							Este criterio es muy relevante

82. **75. ¿La aplicación o las características de la aplicación son personalizables?** *

Marca solo un óvalo.

	0	1	2	3	4	5	
Sugiero eliminar este criterio							Este criterio es muy relevante

83. **76. ¿Necesita siempre usar una conexión activa a Internet para funcionar?** *

Marca solo un óvalo.

	0	1	2	3	4	5	
Sugiero eliminar este criterio							Este criterio es muy relevante

84. **77. ¿Es accesible para personas con problemas de visión u otras discapacidades?** *

Marca solo un óvalo.

	0	1	2	3	4	5	
Sugiero eliminar este criterio							Este criterio es muy relevante

85. **78. ¿La App contempla opciones de uso para personas zurdas?** *

Marca solo un óvalo.

	0	1	2	3	4	5	
Sugiero eliminar este criterio							Este criterio es muy relevante

86. **79. ¿La App contempla opciones culturalmente relevantes? (Por ejemplo opciones para personas veganas, musulmanes, alfabetos diferentes, etc.)** *

Marca solo un óvalo.

	0	1	2	3	4	5	
Sugiero eliminar este criterio							Este criterio es muy relevante

87. **80. ¿Dispone la App de un medio de contacto para posibles consultas?** *

Marca solo un óvalo.

	0	1	2	3	4	5	
Sugiero eliminar este criterio							Este criterio es muy relevante

88. **a) ¿Echas en falta algún criterio?**

8. Estética

El siguiente bloque recoge el conjunto de criterios relacionados con la estética de la App: gráficos, diseño, atractivo,..

89. **81. La App ¿es atractiva visualmente? ¿es adecuada la combinación de colores y hay consistencia estilística?** *

Marca solo un óvalo.

	0	1	2	3	4	5	
Sugiero eliminar este criterio	◯	◯	◯	◯	◯	◯	Este criterio es muy relevante

90. **82. Diseño: ¿La disposición y el tamaño de los botones, iconos, menús y contenido en la pantalla son apropiados o ampliables si es necesario?** *

Marca solo un óvalo.

	0	1	2	3	4	5	
Sugiero eliminar este criterio	◯	◯	◯	◯	◯	◯	Este criterio es muy relevante

91. **83. ¿Los gráficos utilizados para botones, iconos, menús y contenido son de buena calidad y alta resolución?** *

Marca solo un óvalo.

	0	1	2	3	4	5	
Sugiero eliminar este criterio	◯	◯	◯	◯	◯	◯	Este criterio es muy relevante

92. **84. ¿El repintado de la pantalla de la App funciona correctamente en cambios de orientación del dispositivo, menús emergentes, ventanas emergentes, etc.?** *

Marca solo un óvalo.

	0	1	2	3	4	5	
Sugiero eliminar este criterio	◯	◯	◯	◯	◯	◯	Este criterio es muy relevante

93. **85. ¿ El tamaño de los elementos interactivos es idóneo?.** *

Marca solo un óvalo.

	0	1	2	3	4	5	
Sugiero eliminar este criterio	◯	◯	◯	◯	◯	◯	Este criterio es muy relevante

94. **a) ¿Echas en falta algún criterio?**

9. Nivel de desarrollo de la App

El siguiente bloque recoge el conjunto de criterios relacionados con el nivel de desarrollo de la App y su interoperabilidad

95. **86. ¿Es el usuario el propietario de los datos?** *

Marca solo un óvalo.

	0	1	2	3	4	5	
Sugiero eliminar este criterio	⬭	⬭	⬭	⬭	⬭	⬭	Este criterio es muy relevante

96. **87. ¿Es el proveedor o desarrollador el propietario de los datos?** *

Marca solo un óvalo.

	0	1	2	3	4	5	
Sugiero eliminar este criterio	⬭	⬭	⬭	⬭	⬭	⬭	Este criterio es muy relevante

97. **88. ¿La App puede compartir datos con la Historia Clinica Electrónica?** *

Marca solo un óvalo.

	0	1	2	3	4	5	
Sugiero eliminar este criterio	⬭	⬭	⬭	⬭	⬭	⬭	Este criterio es muy relevante

98. **89. ¿La App permite imprimir /exportar/descargar tus datos?** *

Marca solo un óvalo.

	0	1	2	3	4	5	
Sugiero eliminar este criterio	⬭	⬭	⬭	⬭	⬭	⬭	Este criterio es muy relevante

99. **90. ¿La App puede compartir datos con otras herramientas de datos de usuario (por ejemplo, Apple HealthKit, FitBit)?** *

Marca solo un óvalo.

	0	1	2	3	4	5	
Sugiero eliminar este criterio	⬭	⬭	⬭	⬭	⬭	⬭	Este criterio es muy relevante

100. **91. ¿La App esta disponible en iOS y Android?** *
Marca solo un óvalo.

	0	1	2	3	4	5	
Sugiero eliminar este criterio	⬭	⬭	⬭	⬭	⬭	⬭	Este criterio es muy relevante

101. **a) ¿Echas en falta algún criterio?**

10. Indicadores de Salud. Datos personales

En esta sección se abordarán los aspectos más relevantes relacionados con el control de sobrepeso y obesidad

102. **92. ¿Contiene la App opciones para registrar la edad de los usuarios?** *
Marca solo un óvalo.

	0	1	2	3	4	5	
Sugiero eliminar este criterio	⬭	⬭	⬭	⬭	⬭	⬭	Este criterio es muy relevante

103. **93. ¿Contiene la App opciones para registrar el género del usuario?** *
Marca solo un óvalo.

	0	1	2	3	4	5	
Sugiero eliminar este criterio	⬭	⬭	⬭	⬭	⬭	⬭	Este criterio es muy relevante

104. **94. ¿Contiene la App opciones para registrar grupo sanguíneo del usuario?** *
Marca solo un óvalo.

	0	1	2	3	4	5	
Sugiero eliminar este criterio	⬭	⬭	⬭	⬭	⬭	⬭	Este criterio es muy relevante

105. **95. ¿Contiene la App opciones para registrar alergias del usuario?** *
Marca solo un óvalo.

	0	1	2	3	4	5	
Sugiero eliminar este criterio	⬭	⬭	⬭	⬭	⬭	⬭	Este criterio es muy relevante

106. **96. ¿Contiene la App opciones para registrar antecedentes personales del usuario relacionados con su salud?** *
Marca solo un óvalo.

	0	1	2	3	4	5	
Sugiero eliminar este criterio	◯	◯	◯	◯	◯	◯	Este criterio es muy relevante

107. **97. ¿Contiene la App opciones para registrar antecedentes familiares del usuario relacionados con su salud?** *
Marca solo un óvalo.

	0	1	2	3	4	5	
Sugiero eliminar este criterio	◯	◯	◯	◯	◯	◯	Este criterio es muy relevante

108. **a) ¿Echas en falta algún criterio?**

11. Indicadores de Salud. Datos de estado físico

En esta sección se abordarán aspectos que tienen que ver con el estado físico del usuario

109. **98. ¿Contiene la App opciones para registrar la altura del usuario?** *
Marca solo un óvalo.

	0	1	2	3	4	5	
Sugiero eliminar este criterio	◯	◯	◯	◯	◯	◯	Este criterio es muy relevante

110. **99. En el caso de que la App contenga opciones para registrar la altura del usuario ¿puede realizarse de modo progresivo contemplando la evolución en el tiempo?** *
Marca solo un óvalo.

	0	1	2	3	4	5	
Sugiero eliminar este criterio	◯	◯	◯	◯	◯	◯	Este criterio es muy relevante

111. **100. ¿Contiene la App opciones para registrar el peso del usuario?** *
Marca solo un óvalo.

	0	1	2	3	4	5	
Sugiero eliminar este criterio	◯	◯	◯	◯	◯	◯	Este criterio es muy relevante

112. **101. En el caso de que la App contenga opciones para registrar el peso del usuario ¿puede realizarse de modo progresivo contemplando la evolución en el tiempo?** *
Marca solo un óvalo.

	0	1	2	3	4	5	
Sugiero eliminar este criterio							Este criterio es muy relevante

113. **102. ¿Contiene la App opciones para registrar dimensiones corporales del usuario? (medidas de cintura, cadera o muslo)** *
Marca solo un óvalo.

	0	1	2	3	4	5	
Sugiero eliminar este criterio							Este criterio es muy relevante

114. **103. En el caso de que la App contenga opciones para registrar dimensiones corporales del usuario ¿puede realizarse de modo progresivo contemplando la evolución en el tiempo?** *
Marca solo un óvalo.

	0	1	2	3	4	5	
Sugiero eliminar este criterio							Este criterio es muy relevante

115. **104. ¿Realiza la App el calculo del Indice de Masa Corporal?** *
Marca solo un óvalo.

	0	1	2	3	4	5	
Sugiero eliminar este criterio							Este criterio es muy relevante

116. **105. En el caso de que la App realice el cálculo del Indice de Masa Corporal ¿puede realizarse de modo progresivo contemplando la evolución en el tiempo?** *
Marca solo un óvalo.

	0	1	2	3	4	5	
Sugiero eliminar este criterio							Este criterio es muy relevante

117. **106. ¿Contiene la App opciones para registrar el historial diabético del usuario?** *
Marca solo un óvalo.

	0	1	2	3	4	5	
Sugiero eliminar este criterio							Este criterio es muy relevante

118. **107. ¿Contiene la App opciones para registrar el nivel de colesterol del usuario?** *
Marca solo un óvalo.

	0	1	2	3	4	5	
Sugiero eliminar este criterio	⬭	⬭	⬭	⬭	⬭	⬭	Este criterio es muy relevante

119. **108. ¿Contiene la App opciones para registrar el nivel de triglicéridos del usuario?** *
Marca solo un óvalo.

	0	1	2	3	4	5	
Sugiero eliminar este criterio	⬭	⬭	⬭	⬭	⬭	⬭	Este criterio es muy relevante

120. **109. ¿Permite la App estimar el nivel de riesgo cardiovascular del usuario en base a los datos previos?** *
Marca solo un óvalo.

	0	1	2	3	4	5	
Sugiero eliminar este criterio	⬭	⬭	⬭	⬭	⬭	⬭	Este criterio es muy relevante

121. **110. ¿Contiene la App opciones para registrar la presión arterial del usuario?** *
Marca solo un óvalo.

	0	1	2	3	4	5	
Sugiero eliminar este criterio	⬭	⬭	⬭	⬭	⬭	⬭	Este criterio es muy relevante

122. **111. ¿Contiene la App opciones para registrar el pulso en reposo del usuario?** *
Marca solo un óvalo.

	0	1	2	3	4	5	
Sugiero eliminar este criterio	⬭	⬭	⬭	⬭	⬭	⬭	Este criterio es muy relevante

123. **112. ¿Contiene la App opciones para registrar tratamientos farmacológicos seguidos por el usuario?** *
Marca solo un óvalo.

	0	1	2	3	4	5	
Sugiero eliminar este criterio	⬭	⬭	⬭	⬭	⬭	⬭	Este criterio es muy relevante

124. **a) ¿Echas en falta algún criterio?**

12. Indicadores de salud. Datos de actividad

En esta sección se abordan los aspectos relativos a los hábitos relacionados con la salud de los usuarios

125. **113. ¿Contiene la App opciones para registrar el tipo de actividad física realizada por el usuario?** *

Marca solo un óvalo.

	0	1	2	3	4	5	
Sugiero eliminar este criterio	◯	◯	◯	◯	◯	◯	Este criterio es muy relevante

126. **114. ¿Contiene la App opciones para registrar la cantidad de actividad física realizada por el usuario?** *

Marca solo un óvalo.

	0	1	2	3	4	5	
Sugiero eliminar este criterio	◯	◯	◯	◯	◯	◯	Este criterio es muy relevante

127. **115. ¿Contiene la App opciones para registrar la frecuencia de la actividad física realizada por el usuario?** *

Marca solo un óvalo.

	0	1	2	3	4	5	
Sugiero eliminar este criterio	◯	◯	◯	◯	◯	◯	Este criterio es muy relevante

128. **116. ¿Contiene la App opciones para registrar la distancia recorrida por el usuario o el número de pasos que hace?** *

Marca solo un óvalo.

	0	1	2	3	4	5	
Sugiero eliminar este criterio	◯	◯	◯	◯	◯	◯	Este criterio es muy relevante

129. **117. ¿Contiene la App opciones para calcular el gasto calórico que realiza el usuario?** *

Marca solo un óvalo.

	0	1	2	3	4	5	
Sugiero eliminar este criterio	◯	◯	◯	◯	◯	◯	Este criterio es muy relevante

130. **118. ¿Contiene la App opciones para registrar el tipo de dieta que sigue el usuario?** *
Marca solo un óvalo.

	0	1	2	3	4	5	
Sugiero eliminar este criterio	⬭	⬭	⬭	⬭	⬭	⬭	Este criterio es muy relevante

131. **119. ¿Contiene la App opciones para registrar la cantidad de alimentos que consume el usuario?** *
Marca solo un óvalo.

	0	1	2	3	4	5	
Sugiero eliminar este criterio	⬭	⬭	⬭	⬭	⬭	⬭	Este criterio es muy relevante

132. **120. ¿Contiene la App opciones para registrar la frecuencia de consumo de alimentos del usuario?** *
Marca solo un óvalo.

	0	1	2	3	4	5	
Sugiero eliminar este criterio	⬭	⬭	⬭	⬭	⬭	⬭	Este criterio es muy relevante

133. **121. ¿Contiene la App opciones para registrar el consumo de tabaco del usuario?** *
Marca solo un óvalo.

	0	1	2	3	4	5	
Sugiero eliminar este criterio	⬭	⬭	⬭	⬭	⬭	⬭	Este criterio es muy relevante

134. **122. ¿Contiene la App opciones para registrar el consumo de alcohol del usuario?** *
Marca solo un óvalo.

	0	1	2	3	4	5	
Sugiero eliminar este criterio	⬭	⬭	⬭	⬭	⬭	⬭	Este criterio es muy relevante

135. **123. ¿Contiene la App opciones para registrar el consumo de otros tóxicos del usuario?** *
Marca solo un óvalo.

	0	1	2	3	4	5	
Sugiero eliminar este criterio	⬭	⬭	⬭	⬭	⬭	⬭	Este criterio es muy relevante

136. **124. ¿Contiene la App opciones para registrar las horas de sueño del usuario?** *
Marca solo un óvalo.

	0	1	2	3	4	5	
Sugiero eliminar este criterio	⬭	⬭	⬭	⬭	⬭	⬭	Este criterio es muy relevante

137. **125. ¿Contiene la App opciones para registrar la calidad del sueño del usuario?** *

Marca solo un óvalo.

	0	1	2	3	4	5	
Sugiero eliminar este criterio	⬭	⬭	⬭	⬭	⬭	⬭	Este criterio es muy relevante

138. **126. ¿Contiene la App opciones para registrar el bienestar emocional del usuario?** *

Marca solo un óvalo.

	0	1	2	3	4	5	
Sugiero eliminar este criterio	⬭	⬭	⬭	⬭	⬭	⬭	Este criterio es muy relevante

139. **127. ¿Contiene la App opciones para registrar el nivel de estrés del usuario?** *

Marca solo un óvalo.

	0	1	2	3	4	5	
Sugiero eliminar este criterio	⬭	⬭	⬭	⬭	⬭	⬭	Este criterio es muy relevante

140. **128. ¿Contiene la App opciones para registrar el nivel de apoyo percibido por parte del entorno familiar y/o laboral del usuario?** *

Marca solo un óvalo.

	0	1	2	3	4	5	
Sugiero eliminar este criterio	⬭	⬭	⬭	⬭	⬭	⬭	Este criterio es muy relevante

141. **a) ¿Echas en falta algún criterio?**

____

____

____

____

____

Se enviará una copia de tus respuestas por correo electrónico a la dirección que has proporcionado

Con la tecnología de Google Forms
