# Supplementary material for: Evaluation Criteria for Weight Management Apps: Validation Using a Modified Delphi Process
Source: JMIR Mhealth Uhealth. 2020 Jul 22;8(7):e16899. doi: 10.2196/16899 (PMC7407251; doi:10.2196/16899)
Supplement: Multimedia Appendix 3 [file mhealth_v8i7e16899_app3.pdf]

MULTIMEDIA APPENDIX 3
LIST OF CRITERIA UNDER CONSENSUS

Purpose of the App

1.1. Does the app clearly describe its purpose (habits promotion, intervention, monitoring)? *

1.2. Accuracy of the application description: Does the application contain what is described (in the app store)? *

1.3. Does the defined purpose of the app correspond to what it actually does? *

Safety and Privacy

2.1. Does the app identify the risks that its management may pose for user safety?

2.2. Does the app correctly manage access to personal information through prior approval by the user? *

2.3. Does the app take into account secure network configurations to prevent data interception, with specific security safeguard measures for mobile networks? *

2.4. In the event that the app uses cloud services, does it declare the terms and conditions of use and guarantee safety? *

2.5 If a device is lost or stolen, can the user inform the provider and block access to the device?

2.6. Can unauthorized access attempts be registered and reported immediately to the data controller?

2.7. At the request of the owner, can the provider delete the app and any related data in the tracking system and documentation of access to the data to avoid any unauthorized access to personal data? *

2.8. Does the app incorporate privacy notices and configuration options?

2.9. Does the app collect only the essential data (according to medical standards) and essential information for its operation?

2.10. Does the app clearly identify the people who carry out actions (changes, deletions and/or incorporations) in the databases?

2.11. Does the app inform users, in a clear and simple way, of legal regulations on personal data protection? *

2.12. Does the app inform users, clearly and simply, about providing data to third parties? *

2.13. Does the app include a privacy policy? *

2.14. Are the user's personal data anonymized?

2.15. Can the user choose not to participate in data transfer?

* Criterion selected

2.16. Can the user erase their data?
2.17. Are cookies added to the device?
2.18. Does the app inform users about what data is shared? *
2.19. Does the app inform users who the data is shared with? *
2.20. Does the app inform users where the data is stored (on the device, on the web, on both)?
2.21. Does the app inform users about what security measures exist? *
2.22. Are the data stored in the app encrypted on the device and the server? *
2.23. Does the app clearly express its adaptation to current regulations relating to privacy and security? *
2.24. Does the app include security aspects relating to adverse effects?

Clinical effectiveness

3.1. Does the app have specific objectives that are appropriate, measurable and attainable (specified in the app store description or within the app)? *
3.2. Is there published and peer-reviewed evidence about the tool?
3.3. Does the app cite and prioritize reliable information sources with scientific evidence (rigor and quality, scientific endorsements, etc.)? *
3.4. If the information sources are not scientifically recognized, is it indicated how the information was prepared and what relevance and reliability the sources have?
3.5. Do the app reviews and updates consist of new scientific evidence? *
3.6. Does the app appear valued with at least a reasonable value in the app store, website, etc.)?
3.7. Does the app use reliable and valid data systems, recognized by medical professionals, specialists, scientific societies and/or health agencies? *
3.8. Are health recommendations offered by the app based on the data collected in accordance with scientific evidence? *
3.9. Does the app present strategies to increase user adherence (such as gamification, the ability to create challenges or rewards, the registration of habits, etc.)?
3.10. Is the app based on some motivational psychological theory?
3.11. Does the app avoid the use of logos or other elements that may lead to conflict of interest?

Reliability

4.1. Is the content of the app correct, well written and relevant to the objective? *
4.2. Credibility: does the app come from a legitimate source (specified in the app store description or within the app itself)? *

* Criterion selected

4.3. Does the clinical information contained in the app have the endorsement of scientific societies or professional associations?
4.4. Does the clinical information contained in the app have the endorsement of any health organization or health authority?
4.5. Does the app warn about inappropriate behaviors or proposals (e.g. when there is a risk of very restrictive diets or when it is necessary to consult a professional)? *
4.6. In the event that the app sells products or services, does it provide clear and understandable information on the conditions of electronic commerce? *
4.7. In the event that the app sells products or services, does it provide clear information on conflicts of interest or ethical statements in this regard?
4.8. Is there evidence that the app has been tested and verified by evidence (in published scientific literature)? *

Usability

5.1. Does the app have a friendly and intuitive interface? *
5.2. Is the app fun/entertaining to use? Does the app use any strategy to increase participation through entertainment (for example, through gamification)?
5.3. Does the app use language relevant for the audience (in general terms, an understandable, plain and simple language, with messages adapted to the user profile in terms of linguistic style and level, facilitating user understanding and avoiding using technicalities)? *
5.4. Are navigation options and relevant content easily perceived by the user at an initial glance? *
5.5. Is navigation between the sections of the app easy, intuitive and fast? *
5.6. Does the app present a friendly design on the data entry forms? *
5.7. Is the functionality of each element clearly identifiable (for example, if the user must take a specific action, the app should clearly and visually indicate the action to be taken)? *
5.8. Is operation of the app fast? *
5.9. Does the app provide information about long-term use?
5.10. Does the app inform users about possible malfunctions?
5.11. Interactivity: Does the app allow user input, provide comments, contain notices (reminders, sharing options, notifications, etc.)? Note: these functions should be customizable and not overwhelming. *
5.12. Is the content of the app (visual information, language, design) appropriate for the target audience?

* Criterion selected

5.13. Does the app use graphic resources (such as contrast or color inversion) to highlight which item or section has been selected and which fields have not been filled in correctly or are incorrect?
5.14. Are gestural design interactions (touches, scrolls, etc.) consistent and intuitive in all components and screens? *
5.15 In the data log changes, does the app introduce help on what information is needed (for example, descriptive, related drop-down, default calendars for dates, etc.)?
5.16. Is the app accessible to people with vision problems or other disabilities?
5.17. Does the app include use options for left-handed people?

Functionality

6.1. Is the app easy to use? *
6.2. Do the functions and components of the app (buttons/menus) work accurately and quickly? *
6.3. Does the app present "freeze" bugs that interrupt your interaction (for example, if the user accepts an incoming call while the app is running, it should be possible to return to the same point at the end of the call)?
6.4. Are menu labels, icons and instructions clear? *
6.5. Are all necessary screen links present?
6.6. Are the app or app features customizable?
6.7. Does the app always need to use an active Internet connection?
6.8. Does the app include culturally relevant options (e.g. options for vegans, Muslims, different alphabets, etc.)?
6.9. Does the app have a means of contact for possible inquiries?

Level of development

7.1. Is it specified who owns the data (the user, the provider, both)? *
7.2. Can the app share data with the Electronic Health Record? *
7.3. Does the app allow users to print/export/download data? *
7.4. Can the app share data with other user data tools (for example, Apple HealthKit, FitBit)?
7.5. Is the app available on iOS and Android? *

Health indicators: Personal data

8.1. Does the app contain options to record the age of users? *
8.2. Does the app include any specificity for use by minors?
8.3. Does the app contain options to register the user's gender?

* Criterion selected

8.4. Does the app contain options to register user allergies?
8.5. Does the app contain options to record the user's personal health history? *
8.6. Does the app contain options to record the user's family health history?

Health indicators: Physical state data

9.1. Does the app contain options to record user height? *
9.2. In the event that the app contains options to record the user's height, can it be done progressively over time?
9.3. Does the app contain options to record the user's weight? *
9.4. In the event that the app contains options to record the user's weight, can it be done progressively over time? *
9.5. Does the app contain options to register user body dimensions (e.g. waist measurements)?
9.6. In the event that the app contains options to register user body dimensions, can it be done progressively over time? *
9.7. Does the app calculate Body Mass Index?
9.8. In the event that the app calculates Body Mass Index, can it be done progressively over time?
9.9. Does the app contain options to record the user's diabetic history?
9.10. Does the app contain options to record the user's cholesterol level?
9.11. Does the app contain options to record the user's triglyceride level?
9.12. Does the app estimate the user's cardiovascular risk level based on previous data?
9.13. Does the app contain options to record the user's blood pressure?
9.14. Does the app contain options to record the user's resting pulse?
9.15. Does the app contain options to register the user’s pharmacological treatments?

Health indicators: Activity data

10.1. Does the app contain options to record the type of physical activity performed by the user? *
10.2. Does the app contain options to record the amount of physical activity performed by the user? *
10.3 Does the app contain options to record the frequency of physical activity performed by the user? *
10.4 Does the app contain options to record the distance traveled by the user or the number of steps taken?

* Criterion selected

10.5. Does the app contain options to calculate the caloric expenditure made by the user?
10.6. Does the app contain options to record the type of diet the user follows?
10.7 Does the app contain options to record the amount of food the user consumes? *
10.8. Does the app contain options to record the frequency of food consumption by the user? *
10.9. Does the app contain options to register the user's tobacco consumption?
10.10. Does the app contain options to record the user's alcohol consumption?
10.11 Does the app contain options to record substance consumption?
10.12. Does the app contain options to record the user's sleep hours?
10.13. Does the app contain options to record the quality of the user's sleep?
10.14. Does the app contain options to record the user's emotional well-being?
10.15 Does the app contain options to record the user's stress level?
10.16. Does the app contain options to record the perceived levels of support from the family and/or work environment of the user?

* Criterion selected
